# Bone marrow-derived mesenchymal stem cells attenuate pulmonary inflammation and lung damage caused by highly pathogenic avian influenza A/H5N1 virus in BALB/c mice

**DOI:** 10.1186/s12879-020-05525-2

**Published:** 2020-11-11

**Authors:** Resti Yudhawati, Muhammad Amin, Fedik A. Rantam, Rima R. Prasetya, Jezzy R. Dewantari, Aldise M. Nastri, Emmanuel D. Poetranto, Laksmi Wulandari, Maria I. Lusida, Soetjipto Koesnowidagdo, Gatot Soegiarto, Yohko K. Shimizu, Yasuko Mori, Kazufumi Shimizu

**Affiliations:** 1 Indonesia-Japan Collaborative Research Center for Emerging and Re-emerging Infectious Diseases, Institute of Tropical Disease, Airlangga University, Surabaya, Indonesia; 2 Department of Pulmonology and Respiratory Medicine, Faculty of Medicine, Airlangga University, Surabaya, Indonesia; 3 Department of Virology and Immunology, Faculty of Veterinary Medicine / Stem Cell Research and Development Center, Airlangga University, Surabaya, Indonesia; 4 Center for Infectious Diseases, Kobe University Graduate School of Medicine, Kobe, Japan

**Keywords:** Acute lung injury, Acute respiratory distress syndrome, Bone marrow-derived mesenchymal stem cells, Highly pathogenic avian influenza a/H5N1 virus, Cell-based therapy, BALB/c mouse, Inflammatory cytokines, Arterial blood gas analysis, Alveolar cell regeneration

## Abstract

**Background:**

The highly pathogenic avian influenza A/H5N1 virus is one of the causative agents of acute lung injury (ALI) with high mortality rate. Studies on therapeutic administration of bone marrow-derived mesenchymal stem cells (MSCs) in ALI caused by the viral infection have been limited in number and have shown conflicting results. The aim of the present investigation is to evaluate the therapeutic potential of MSC administration in A/H5N1-caused ALI, using a mouse model.

**Methods:**

MSCs were prepared from the bone marrow of 9 to 12 week-old BALB/c mice. An H5N1 virus of A/turkey/East Java/Av154/2013 was intranasally inoculated into BALB/c mice. On days 2, 4, and 6 after virus inoculation, MSCs were intravenously administered into the mice. To evaluate effects of the treatment, we examined for lung alveolar protein as an indicator for lung injury, PaO_2_/FiO_2_ ratio for lung functioning, and lung histopathology. Expressions of NF-κB, RAGE (transmembrane receptor for damage associated molecular patterns), TNFα, IL-1β, Sftpc (alveolar cell type II marker), and Aqp5+ (alveolar cell type I marker) were examined by immunohistochemistry. In addition, body weight, virus growth in lung and brain, and duration of survival were measured.

**Results:**

The administration of MSCs lowered the level of lung damage in the virus-infected mice, as shown by measuring lung alveolar protein, PaO_2_/FiO_2_ ratio, and histopathological score. In the MSC-treated group, the expressions of NF-κB, RAGE, TNFα, and IL-1β were significantly suppressed in comparison with a mock-treated group, while those of Sftpc and Aqp5+ were enhanced. Body weight, virus growth, and survival period were not significantly different between the groups.

**Conclusion:**

The administration of MSCs prevented further lung injury and inflammation, and enhanced alveolar cell type II and I regeneration, while it did not significantly affect viral proliferation and mouse morbidity and mortality. The results suggested that MSC administration was a promissing strategy for treatment of acute lung injuries caused by the highly pathogenic avian influenza A/H5N1 virus, although further optimization and combination use of anti-viral drugs will be obviously required to achieve the goal of reducing mortality.

## Background

Acute lung injury (ALI) can cause acute respiratory failure, namely acute respiratory distress syndrome (ARDS) [1, 2]. ARDS is characterized by diffuse alveolar damage, which ultimately results in severe hypoxemia and respiratory failure [1, 3]. Infection with the highly pathogenic avian influenza A/H5N1 virus is one of the causes for ARDS [4, 5]. Since antiviral medicines cannot repair tissue injury caused by influenza viruses [6], cell-based therapy is a potential new therapeutic approach. Mesenchymal stem cells (MSCs) possess the ability to regulate hematopoietic cells, secreting several regulatory molecules such as growth factors and anti-inflammatory cytokines, which are able to modulate immune responses [7]. Studies of the role of MSCs in ALI induced by influenza A viruses have yielded conflicting results to date [8–12].

Injury of alveolar epithelial-endothelial barrier cells is the main cause of ARDS. The damage causes a leak of protein from intravascular into alveolar lumen [3, 13]. It is mentioned in the literature that injury of alveolar epithelial-endothelial barrier cells occurs due to immune system dysregulation [5, 13]. Immune system dysregulation is one of the key mechanisms in the pathogenesis of influenza A viruses [14, 15]. Pathogen-associated molecular patterns (PAMPs) during the viral infection are identified by three main classes of pattern recognition receptors (PRRs) [16]. These three pathways can induce production of proinflammatory cytokines and chemokines, which cause lung damage [16, 17]. The TLR7 and RIG-I signals through adaptor proteins will activate nuclear factor-κB (NF-κB) which acts as a transcription factor inducing production of the proinflammatory cytokines [17, 18]. Besides PAMPs, danger-associated molecular patterns (DAMPs) are host molecules functioning to regulate activation of PRRs. The high mobility group box 1 (HMGB1) is a DAMP released from damaged cells [19, 20]. HMGB1 binding with receptor for advanced glycation end products (RAGE) gives a signal which activates NF-κB and then induces adhesion molecules and proinflammatory cytokines [21, 22]. Through the NF-κB pathway, influenza A virus will also activate proapoptotic factors, namely TNF-related apoptosis-inducing ligand (TRAIL) and FasL [23]. This next activates caspases [24]. Caspase activation results in increase of nucleic exports of viral ribonucleoprotein and regulates viral RNA synthesis [25]. The administration of MSCs allotransplantation is expected to produce a beneficial effect on tissue injury through an inhibition mechanism against the NF-κB transcription factor [26].

Alveolar epithelial cells are pulmonary endogenous progenitor cells which are damaged by viral infection [27]. MSCs are multipotent cells that can differentiate into cell lineage of mesodermal, endodermal and ectodermal cells [28]. Type II alveolar epithelial cells can renew themselves and act as progenitor cells for type I alveola epithelial cells during normal homeostatic as well as after epithelial damage due to injury [29]. In addition, the administration of MSCs allotransplantation is also expected to act as an immune regulator to maintain cellular homeostasis stimulating endogenous MSCs and pulmonary epithelial cell progenitors to renew themselves.

Mouse models have been useful to investigate the role of MSCs in ALI induced by influenza A viruses, although conflicting results have been obtained [8, 10–12]. BALB/c mice can be infected by avian influenza viruses, accompanied by pathological changes, because α-2,3 sialic acid receptors are present on their airway ciliated epithelial cells and type II alveolar epithelial cells, which avian influenza viruses recognize [30]. In addition, it was reported that responses of BALB/c mice to the virus infection had some similarities to those of humans, especially in terms of host immune response [31–33].

In Indonesia, highly pathogenic avian influenza A/H5N1 virus has been endemic in poultry since 2003 and causes sporadic infection in humans. Indonesia is a country with high cumulative number of human infections with the virus, recording 200 cases with 168 mortalities during 2003 to 2019, which was the highest mortality rate in the world [34]. Viruses of H5 HA clade 2.1 had been exclusively circulating in poultry until 2012 in Indonesia. Incursion of viruses of clade 2.3.2.1c was reported for the first time in September 2012 [35]. In September 2013, we isolated a virus of clade 2.3.2.1c, A/turkey/East Java/Av154/2013, from an outbreak at a turkey farm in East Java, Indonesia [36]. This virus was highly virulent and lethal in mice [37]. H5N1 viruses of only clade 2.3.2.1c are currently circulating in poultry in East Java (unpublished data).

Therefore, using the virus and BALB/c mouse model, we aimed to investigate the effect of MSCs on ALI induced by infection of a highly pathogenic avian influenza virus in terms of suppression of lung injury and inflammation, and enhancement of alveolar cell type II and I regeneration. We examined for lung alveolar protein as an indicator of lung injury, PaO_2_/FiO_2_ ratio as an indicator of lung functioning, and lung histopathology. Expressions of NF-κB, RAGE, tumor necrosis factor-α (TNF-α), interleukin-1β (IL-1β), surfactant protein-C (Sftpc, alveolar cell type II marker), and aquaporin 5 (Aqp5+, alveolar cell type I marker) were examined by immunohistochemistry. Body weight, virus growth in lung and brain, and duration of survival were also measured.

## Methods

### Experimental design

**Fig. 1:**
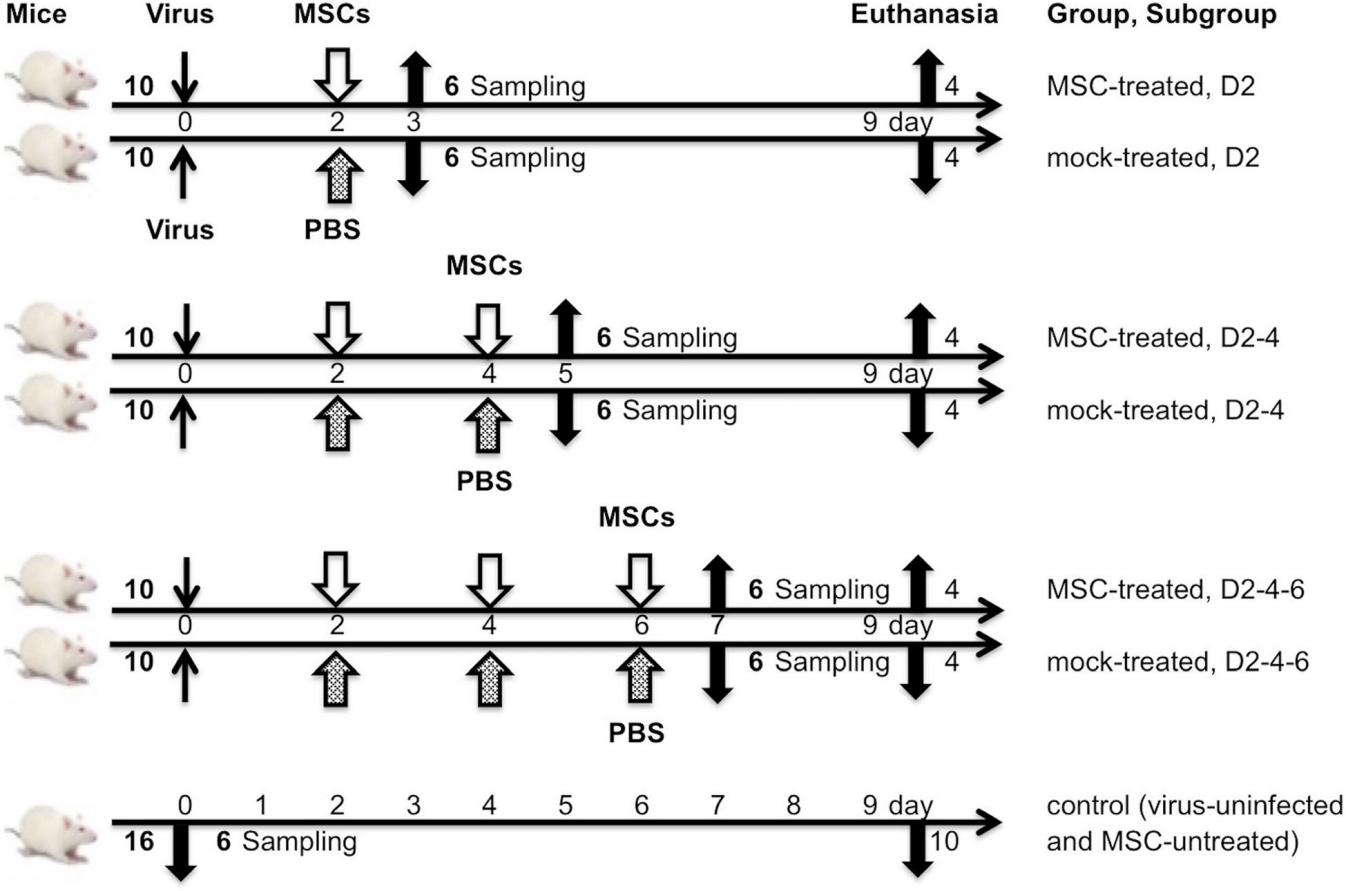
The timeline for virus inoculation and MSC administration in the mouse experiments. Seventy-six BALB/c mice were used in this study. The animals were divided into three groups; MSC-treated group (*n* = 30), mock-treated group (n = 30), and control group (*n* = 16). All mice except for the control group were intranasally inoculated with a dose of 500 MLD50 of a highly pathogenic avian influenza A/H5N1 virus on day 0. The MSC -treated group was further divided into 3 subgroups of D2, D2–4, and D2–4-6 (each group, *n* = 10); subgroup D2 received intravenously 100 μL phosphate buffered saline (PBS) containing 5.5 × 105 of MSCs on day 2, and six mice were terminated day 3 for sampling. Subgroup D2–4 received MSCs on days 2 and 4, and six mice were terminated on day 5. Subgroup D2–4-6 received MSCs on days 2, 4, and 6, and six mice were terminated on day 7. The mock-treated group was a therapy control group with virus infection that received 100 μL of PBS without MSCs. This group was also divided into 3 subgroups of D2, D2–4, and D2–4-6. With the same schedule as for the MSC-treated subgroups, they received PBS and were terminated. The control group was a virus-uninfected and MSC-untreated group. Mice in the control group were terminated on day 0 (*n* = 6) after being randomly selected. All mice were subjected to daily measurement of body weight and examination for survival until day 9. All surviving mice on day 9 were subjected to euthanasia using intraperitoneal injection of high dose ketamine and xylasin

Seventy-six BALB/c mice were used in this study. The animals were divided into three groups; MSC-treated group (*n* = 30), mock-treated group (n = 30), and control group (*n* = 16). Figure 1 ilustrates the timeline for virus inoculation and MSC administration. All mice except the control group were intranasally inoculated with a dose of 500 MLD_50_ of a highly pathogenic avian influenza A/H5N1 virus on day 0. The MSC -treated group was further divided into 3 subgroups of D2, D2–4, and D2–4-6 (each group, *n* = 10); subgroup D2 received intravenously 100 μL phosphate buffered saline (PBS) containing 5.5 × 10^5^ of MSCs on day 2, and six mice were terminated day 3 for sampling. Subgroup D2–4 received MSCs on days 2 and 4, and six mice were terminated on day 5. Subgroup D2–4-6 received MSCs on days 2, 4, and 6, and six mice were terminated on day 7. The mock-treated group was a therapy control group with virus infection that received 100 μL of PBS without MSCs. This group was also divided into 3 subgroups of D2, D2–4, and D2–4-6. With the same schedule as for the MSC-treated subgroups, they received PBS and were terminated. The control group was a virus-uninfected and MSC -untreated group. Mice in the control group were terminated on day 0 (*n* = 6) after being randomly selected. All mice were subjected to daily measurement of body weight and examination for survival until day 9. The sample size was determined following the method described by Lemeshow et al. (1990) [38] using variant values obtained for TNFα level in the preliminary studies. Although the minimum number of mice in each group was calculated to be at least 5, 20% was added to be 6 in this study for estimation of failure.

### Ethics statement

This study was carried out in strict accordance with the recommendations in the Guide for the Care and Use of Laboratory Animals of the National Institutes of Health. The Animal Care and Use Committee (ACUC), Faculty of Veterinary Medicine, Universitas Airlangga approved this study; the document identifier is 515-KE. All treatments and surgery were performed under ketamine and xylasin anesthesia to minimize suffering.

### Mice

BALB/c mice used in this study were obtained from Stem Cell Research and Development Center, Airlangga University. The mice (male, 9–12 weeks old) with body weight of 25–30 g have been raised with standard feed and sterile water. Each cage is made of plastic covered with wire mesh filter and has a standard size with floor area of 1200 cm^2^ and height of 25 cm. The number of the cage companions was less than 10. The room temperature was 18–26 °C and the relative humidity was 40–70%. Wood powder was used for the cage floor and cleaned with antiseptic once every 3 days. The cage was placed in a ventilated microisolator enclosure under negative pressure with HEPA-filter air within a biosafety cabinet located in the BSL3 laboratory. Each mouse was fed ad libitum 30 mg/day according to standard procedure with composition of protein 18–20%, fat 5–12%, fiber 2.5%, and carbohydrate 60–70%. Water was given by the drip method to avoid contamination with dirt. Before treatment, mice were adapted for one week to their new environment with a dark and light cycle every 12 h. Mice were randomly distributed into the three groups: MSC-treated, mock-treated, and control groups by using simple random technique. The mice not in the control group were further randomly divided into three subgroups for three different schedules of treatment. Each mouse in a given cage was differentiated by ear punching on mice’s ear in different locations and numbers. After experiments, all surviving mice were euthanized by injection of high dose ketamine (450 mg/kg body weight) and xylasin (50 mg/kg bodyweight) intraperitoneally that induces rapid loss of consciousness and death with a minimum of pain, discomfort or distress [39]. We confirmed death of the mice with physical methods; we found 1) absence of heart beat through direct cardiac inspection and palpation, 2) lack of spontaneous breathing by respiratory pattern observations, and 3) the pupils dilated and unresponsiveness to light by checking pupils.

### Bone marrow-derived mesenchymal stem cells (MSCs)

We prepared MSCs following a protocol described by Soleimani and Nadri (2009) [40]. MSCs were isolated from bone marrow of male BALB/c mice, aged 9–12 weeks. The bone marrow was aspirated until reached to 2 mL from the middle part of the femoral intercondylar of both legs. The obtained bone marrow was slowly resuspended with PBS followed by centrifugation at 1600 rpm for 25 min in Ficoll. Four layers consisting of red blood cells, ficoll, buffy coat, and plasma were formed in the tube. The fraction of the buffy coat was taken, mixed with PBS, and recentrifuged at the same speed for 10 min. The pellet was resuspended in growth medium α (Gibco, USA) and cultured in petri dishes at 37^o^ C in a 5% CO_2_ incubator. Growing MSCs attached to the 10 cm diameter petri dish was passaged at 80% confluent, which was repeated 5 times until we obtained enough cells for the experiments. The identification of obtained MSCs was carried out in accordance with the International Society of Cellular Therapy Criteria [41], namely cells are attached to the petri dishes, positive for CD105, and negative for CD45. For identification by flow cytometry, a total of 5 × 10^5^ cells were processed using FITC-conjugated anti-mouse CD45 antibody (BioLegend, USA) and PE-conjugated anti-mouse CD105 antibody (BioLegend, USA). For identification by immunocytochemistry, the cells attached to the dishes during 1 h incubation at 37^o^ C were fixated with 10% formaldehyde for 15 min, washed with PBS containing tween-20, and stained with FITC conjugated anti-mouse CD45 and CD 105 antibodies for 1 h at 37^O^ C. The cells expressing CD45 and CD105 proteins were observed under a fluorescent microscope. MSCs prepared with this protocol have been shown to be pluripotent [40]. The cultured MSCs were suspended in PBS to be 5.5 × 10^6^ cells/ mL and an aliquate of 0.1 mL was administered via tail vein. When homing of MSCs were monitored, the monolayer of MSCs was subjected to labeling of live cells with PKH26 (Sigma-Aldrich, USA) which emits yellowish-green fluorescence for the identification without affecting their function [42, 43].

### Virus inoculation

BALB/c mice were lightly anesthetized with ketamine (90 mg/kg body weight) and xylasin (10 mg/kg body weight) intraperitoneally and then inoculated intranasally with A/turkey/East Java/Av154/2013 (H5N1, H5 HA clade 2.3.2.1c), one of our highly pathogenic avian influenza A/H5N1 isolates [36] (the gene sequences were submitted to GISAID database [44] with isolate ID: EPI_ISL_307002). An optimal viral dose of 500 MLD_50_ (50% mice lethal dose) was used in this study. In our preliminary experiments using this dose, there was a clear histopathological symptom of respiratory disease and acute lung injury on day 2 after virus inoculation (data not shown). The virus diluted in 50 μL of TGS (25 mM Tris–HCl, 140 mM NaCl, 5 mM KCl, 0.7 mM Na_2_HPO_4_-12H_2_O, 5.6 mM glucose, pH 7.4) containing 0.2% bovine serum albumin was gradually inserted into the nostrils (25 μL in each) using a micropipette. All procedures were carried out in the BSL3 laboratory at the Institute of Tropical Disease, Airlangga University, Surabaya, Indonesia.

### Arterial blood gas analysis

Mice were lightly anesthetized with ketamine-xylasin intraperitoneally. A 300 μl of arterial blood was withdrawn into a heparinized syringe percutaneously from the left ventricular under the condition breathing in room air (FiO_2_ 21%) spontaneously. The blood gas analysis was carried out immediately using GEM 3000 blood gas analyzer (Instrumentation Laboratory, A Werfen Company).

### Bronchoalveolar lavage fluid (BALF)

BALF was collected from mice immediately after termination by injection of high dose ketamine. Briefly, BALF was taken from both lungs through the tracheal cannula by instilling sterile 1 mL of cold PBS, which was successively done 3 times. Obtained BALF was examined for protein content using Sysmex XN 1000 (Instrumentation Laboratory, Sysmex Company).

### Histopathology

After BALF was collected, both lungs were taken. One of them was subjected to histopathological and immunohistochemical examinations and another was used for virus infectivity titration and the quantification of the viral genome. For histopathology, one half of lung was fixed with 10% formalin, embedded in paraffin, sliced and stained with hematoxylin-eosin (HE). The assessment in the HE-stained lung tissues was done by examination for existence of edema, alveolar inflammation, interstitial inflamentation, alveolar hemorrhage, interstitial hemorrhage, atelectasis, and necrosis and hyalin membrane formation. These 7 parameters were scored individually using a scale from 0 to 4, where 0 is for no injury in the field of view; 1, 2, and 3 are for 25, 50, and 75% injury in the field of view, respectively; 4 is for injury in the entire field [45]. The sum of the 7 scores represented the lung injury score (0 to 28). The score of one lung was determined by examining 5 viewing fields of each tissue section sample by light microscopy at ^X^400 magnification.

### Immunohistochemistry staining

The tissue of the other half of lung was fixed with 4x% paraformaldehyde for 24 h, stored in 70% alcohol at 4 °C, paraffin-embedded, sectioned in 5 μm slices, installed on poly-L-lysine-coated slides, dewaxed in xylene, and rehydrated with ethanol rinses. An aliquot of 3% H_2_O_2_ solution was used to inhibit endogenous peroxidase activity. Mouse antibodies against NF-κB, RAGE, TNFα, IL-1β, Sftpc, and Aqp5 + (Novus Biological USA, Ltd) were employed as primary antibodies. As a secondary antibody, biotin-labeled anti-mouse antibody was used following by diaminobenzidine-labeled streptavidin (Novus Biological USA, Ltd.). The slides were examined under the light microscope with a photo-documentation facility and blinded observers quantitated the staining. The percentage of positive cells was determined by observation of 5 viewing fields for each tissue section with a ^X^400 magnification. Positive expression appeared as brownish color on the cell nucleus.

### Virus quantifications

The lung was homogenized in 2 mL of TGS containing 0.2% bovine serum albumin and stored at − 80 °C. Virus infectivity in the homogenates was determined by 50% tissue culture infectious dose (TCID_50_) assay using Madin-Darby canine kidney cells (ATCC® CCL-34™). Quantification of the viral genome was performed by one-step TaqMan real-time RT-PCR using a QuantiTect Probe RT-PCR Kit (Qiagen, Tokyo, Japan) as previously described [36]. Extraction of RNA from the lung homogenates was performed using a Qiamp MinElute Virus Spin Kit (Qiagen, Tokyo, Japan). The reaction mixture was prepared from 5 μL of template RNA, each primer at a final concentration of 0.6 μM, probe 0.1 μM, and QuantiTect probe RT-PCR mix, and then subjected to a one-step assay with an ABI model 7300 Instrument by using the following conditions: (step 1) reverse transcription for 30 min at 50 °C, (step 2) 15 min at 95 °C to activate *Taq* polymerase, and (step 3) 45 cycles of 15 s at 94 °C and 75 s at 56 °C. For detection of M gene of the A/H5N1 virus, we used a set of primers (forward, 5′-CCMAG GTCGA AACGT AYGTT CTCTC TATC-3′; reverse, TGCAG RATYG GTCTT GTCTT TAGCC AYTCCA-3′) and probe (FAM-ATYTC GGCTT TGAGG GGGCC TG-MGB). Virus infectivity and viral genome RNA synthesis in brain were also examined on day 7 post inoculation. The brain was isolated after termination by injection of high dose ketamine. One half of the brain was homogenized in 2 mL of TGS containing 0.2% bovine serum albumin and subjected to the quantifications.

### Statistics

All data were expressed as means ± standard deviation (SD) or ± standard error (SE). Statistical analysis was performed using statistical SPSS software package for Windows, version 17.0 (SPSS, Inc., Chicago, IL). One-way ANOVA was followed by a variance test or a Brown-Forsythe test to determine differences between treatment groups. A *p*-value of less than 0.05 was considered to be statistically significant.

## Results

### Culture and identification of MSCs

**Fig. 2:**
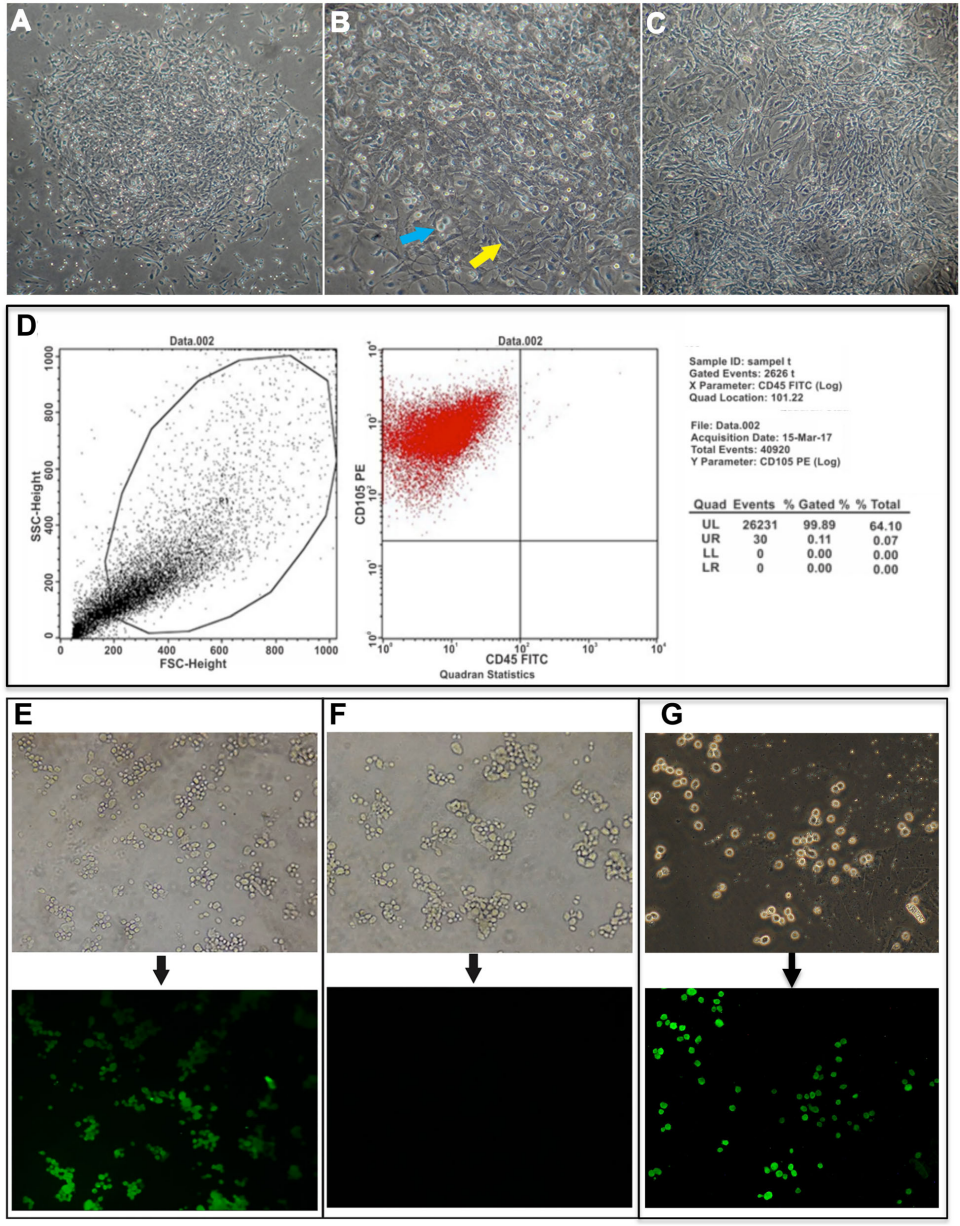
MSCs culture and identification. MSCs were isolated from bone marrow of BALB/c mice as described in Materials and Methods. The cells were suspended in growth medium α (Gibco, USA) and cultured in petri dishes at 37 °C in a 5% CO_2_ incubator. Micrographs of cultured cells on day 11 are shown in **a** with a ^X^100 and **b** with a ^X^400 magnification, and on day 28 in **c** with a ^X^400 magnification. Spindle- or oval-shaped cells like as indicated by a yellow arrow in **b** are MSCs, while round shaped cells like as indicated by a light blue arrow are hematopoietic stem cells which disappeared on day 28. For confirmation of MSCs, the isolated MSCs cells were incubated for 1 h to attach to petri dish and subjected to immunocytochemical analysis. **d** Flow cytometry analysis of the cultured MSCs. Red scattered spots show CD105-positive and CD45-negative cells indicating 99.9% of total cells were MSCs. **e** In the staining with anti-CD105, all of the cells appeared to glow greenish fluorescence under a ^X^100 magnification, indicating that they were positive for CD105. **f** In the staining with anti-CD45, greenish fluorescence was absent, indicating that they were negative for CD45. **g** The ant-CD45 positively stained hematopoietic stem cells provided by Stem Cell Research and Development Center, Airlangga University as a staining-positive control

BALB/c male mice were subjected to MSCs allotransplantation as both donors and recipients in this study. On day 11 culture, several spindle or oval shaped cells (yellow arrow) attached to the petri dish were observed, which were characteristic for MSCs. But, cells were still mixed with hematopoietic stem cells (blue arrow) with a round shape (Fig. 2a and b). After approximately 28 days, fibroblastic spindled shaped cells were found covering the entire petri dish (Fig. 2c). By flow cytometry, 99.9% of cells were CD105-positive and CD45-negative (Fig. 2d). By immunofluorescence, spindle-shaped cells were confirmed to be positive for CD105 (Fig. 2e) and negative for CD45 (Fig. 2f and g).

### Homing of the administrated MSCs to lung

**Fig. 3:**
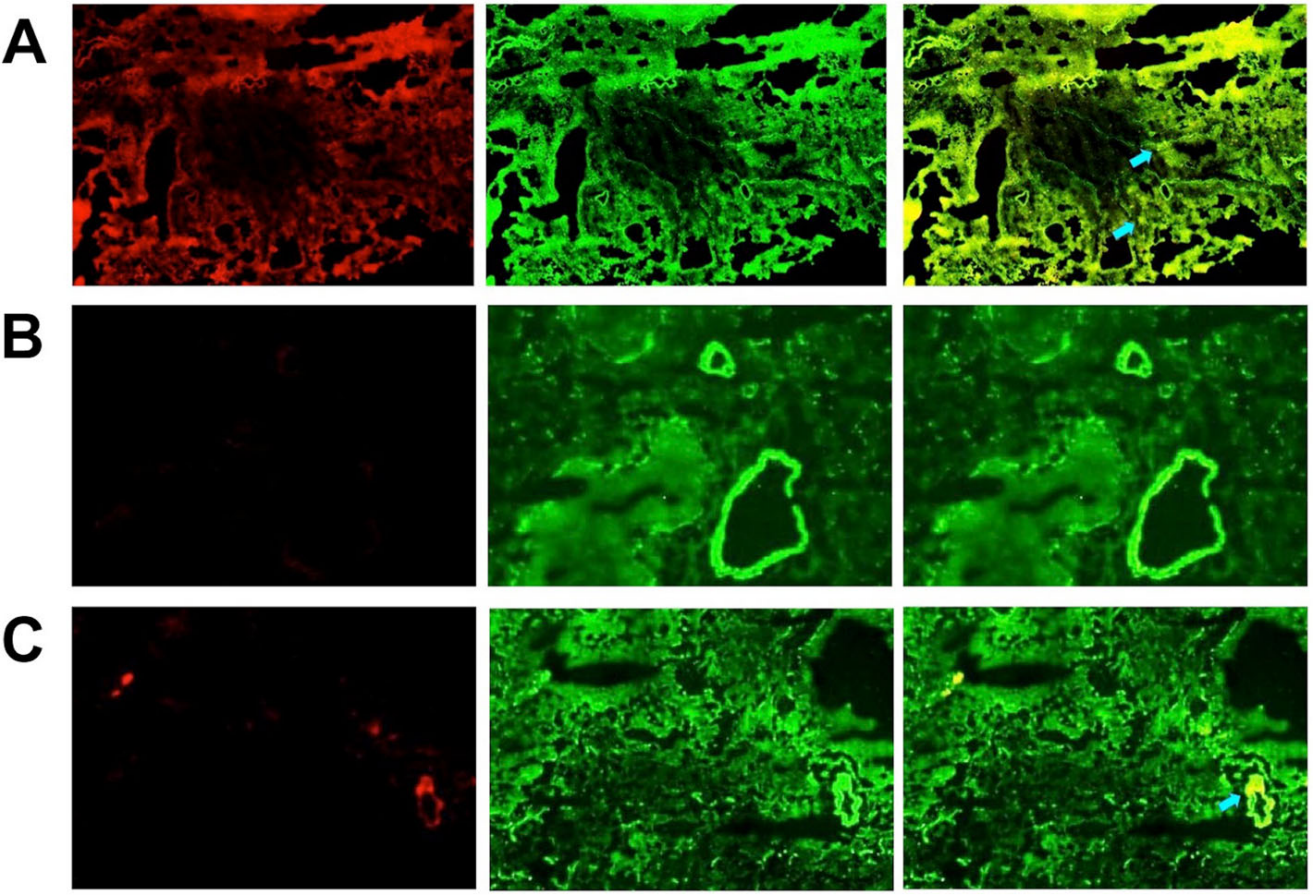
Homing of the administrated MSCs to the lung. The PKH26-labeled MSCs were intravenously administrated via tail vein to mice infected with A/H5N1 virus on day 2 and the mouse lung tissues were examined on day 3 for MSCs homing by detecting fluorescence under the microscope. **a** With MSC administration, the red fluorescence was observed in the entire field of view in the left panel and yellowish green fluorescence in the right panel of merged image, indicating presence of the homing all over the lung. Light blue arrows indicate typical positive spots for yellowish green fluorescence. **b** With control PBS administration, neither red nor yellowish green fluorescence was observed. **c** With MSC administration to normal mice without the virus infection, the red fluorescence was observed in the localized places in the left panel and yellowish green fluorescence localized only on the edges of blood vessels in the right panel of merge image, indicating the absence of MSC homing to the lung tissue. Left panels: PKH26 red fluorescence, center panels: green autofluorescence, and right panels: the merged image of the two

The PKH26-labeled MSCs were intravenously administrated via tail vein to mice infected with A/H5N1 virus on day 2 after inoculation; in our preliminary experiments, clear histopathological signs of lung injury were observed on day 2 (data not shown). The mouse lung tissue was examined on day 3 for MSCs homing by detecting fluorescence under the microscope. One day after administration, the labeled MSCc, were observed to have accumulated throughout the lung tissue, as shown by the yellowish green fluorescence map (Fig. 3a, right) corresponding to the merged images of, respectively, the red fluorescence of labeled MSCs (Fig. 3a, left), and the green autofluorescence of lung tissue (Fig. 3a, center). Light blue arrows indicate typical positive spots for yellowish green fluorescence. As a control, mice of mock-treated group received intravenous PBS. Yellowish green fluorescence was not observed for this group (Fig. 3b). In a preliminary experiment, PKH26-labeled MSCs were administrated to normal mice without virus infection and the yellowish green fluorescence was observed only along the edges of blood vessels in the merged image (Fig. 3c), indicating the absence of MSCs homing to normal lung tissue. These observations evidenced the occurrence of MSC homing to the lung tissue that had acute damage.

### MSCs lowered level of acute lung injury of mice infected with highly pathogenic avian influenza a/H5N1 virus

**Fig. 4:**
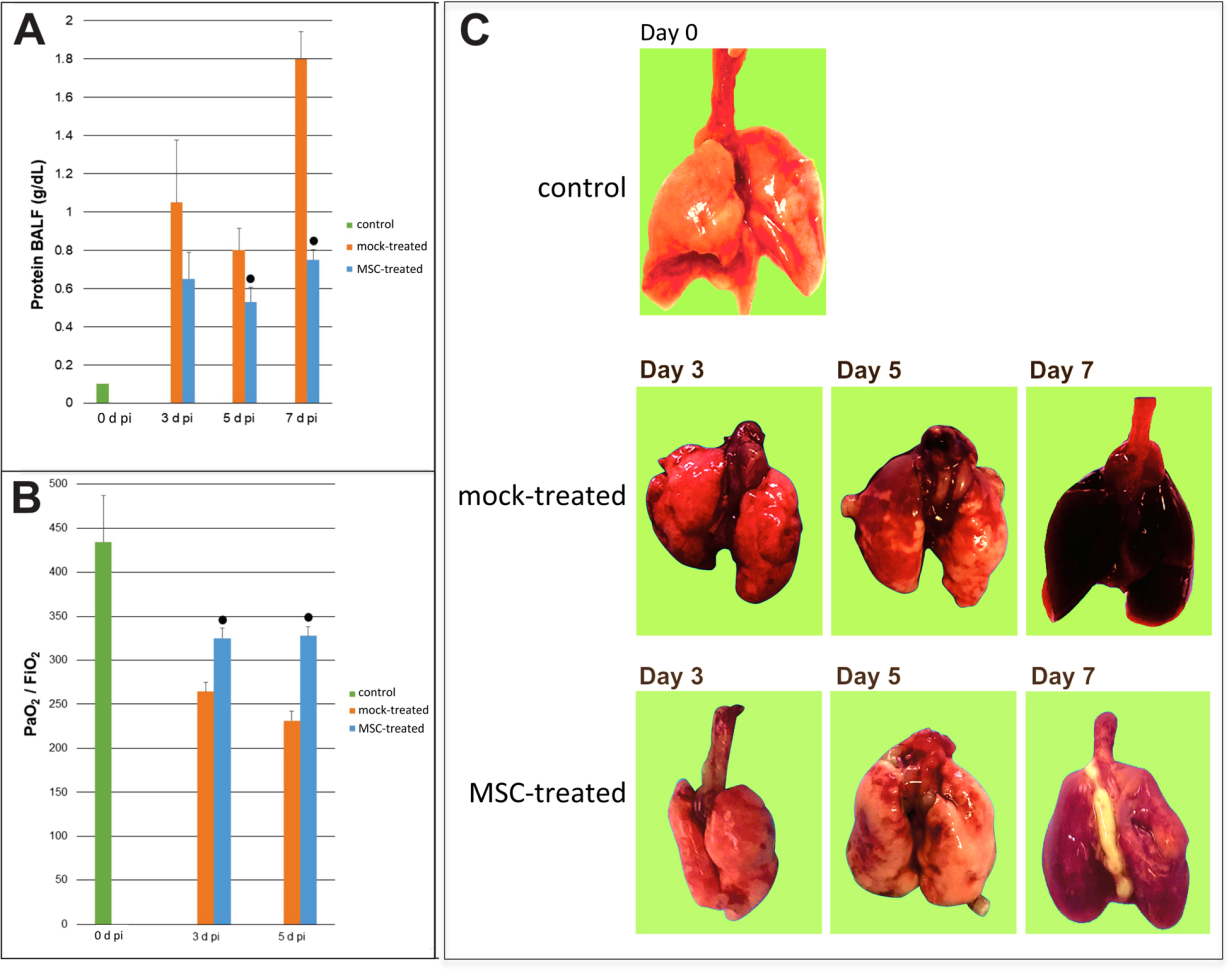
MSCs prevented acute lung injury. The BALF protein (g/dL) and ratio of PaO_2_/FiO_2_ were compared between MSC- and mock-treated groups in **a** and **b**, respectively. Data shown are means ± SD of 6 mice. ● indicates significant difference (*P* < 0.05). d pi: days post-inoculation. Both type of measurements provided quantitative evidence for the reduction of lung damage caused by the virus infection. For reference, the measurements for the control group at day 0 were included. **c** Macroscopic comparison of lung damage between MSC- and mock-treated groups. Extent of lung damage of the MSC-treated group was clearly less than that of the mock-treated group on days 3, 5, and 7

**Fig. 5:**
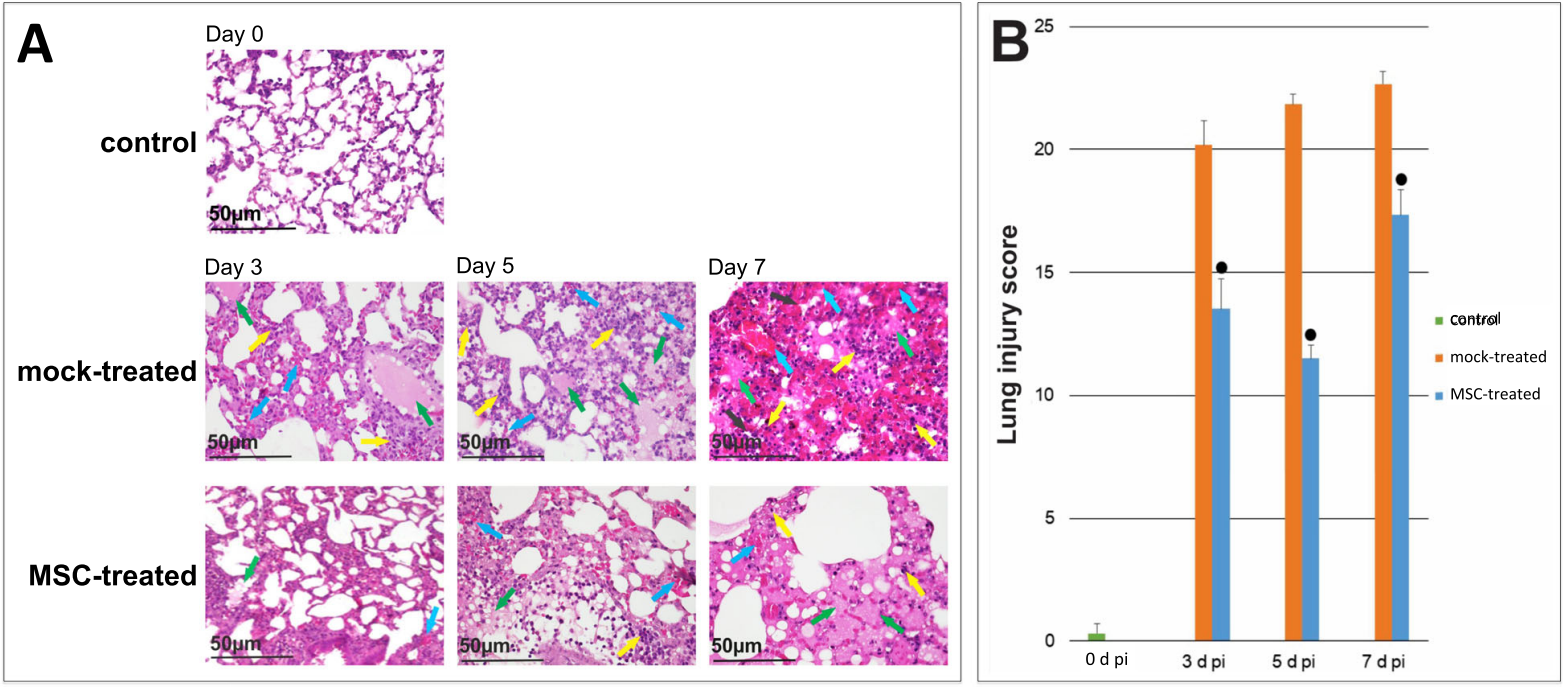
MSCs lowered the level of histological lung injury. **a** Comparison of histopathology for lung tissues by HE staining between MSC- and mock-treated groups. Alveolar edema (green arrows), alveolar inflammation and interstitial tissue (yellow arrows), alveolar bleeding and interstitial tissue (blue arrows), and atelectasis (black arrows) were observed under a light microscopy with a ^X^400 magnification. **b** Comparison of histopathological scores between MSC- and mock-treated groups. Edema, alveolar inflammation, interstitial inflammation, alveolar hemorrhage, interstitial hemorrhage, atelectasis, necrosis and formation of hyaline membranes were individually scored (0 to 4) and the sum of the 7 scores represented the lung injury score (0 to28). Examining 5 viewing fields of each tissue section determined the score of one lung. Data shown are means ± SD of six mice in each group. ● indicates significant difference (P < 0.05) between two groups. d pi: days post-inoculation

Since the leakage of serum proteins into BALF is a quantitative marker of lung injury, we measured the protein contents in BALF of mice (Fig. 4a) and found that the protein leakage occurred from day 3 in mice with the A/H5N1 infection (mock-treated group). The leakage levels were lowered significantly (*p* < 0.05) by the MSC administration; the suppression rates were calculated to be 39% on day 3, 37% on day 5, and 59% on day 7 post-inoculation. We also examined for arterial blood gas and calculated the PaO_2_/FiO_2_ ratio as an indicator of lung functioning on days 3 and 5, but not on day 7 since most of the mice died on day 7 and the surviving mice showed a level of hemolysis that was unacceptably high for the measurement. As shown in Fig. 4b, the ratio dropped from 429 to 259 (60%) on day 3 and 224 (52%) on day 5 in mice with the A/H5N1 lethal infection. When MSCs were administered, the ratios improved significantly: 318 (74%) on day 3 and 324 (76%) on day 5. Both measurements provided quantitative evidence for suppression of lung damage caused by the viral nfection. In preliminary experiments, macroscopic observations revealed obvious injury of lung tissue in the virus-infected mice on day 2 (data not shown). The observations also revealed that the extent of lung damage of the MSC-treated group was clearly lower than that of the mock-treated group on days 3, 5, and 7 (Fig. 4c). Histological examinations of lung tissues by HE staining also showed that MSCs administration suppressed the occurrence of alveolar edema, inflammation and bleeding, interstitial tissue, and atelectasis (Fig. 5a). The histopathological scores were lowered significantly by the MSC treatment; the suppression rate was 33% on day 3, 47% on day 5, and 23% on day 7 (Fig. 5b).

### MSCs attenuated pro-inflammatory signaling and cytokines in the lungs of mice with the a/H5N1 lethal infection

**Fig. 6:**
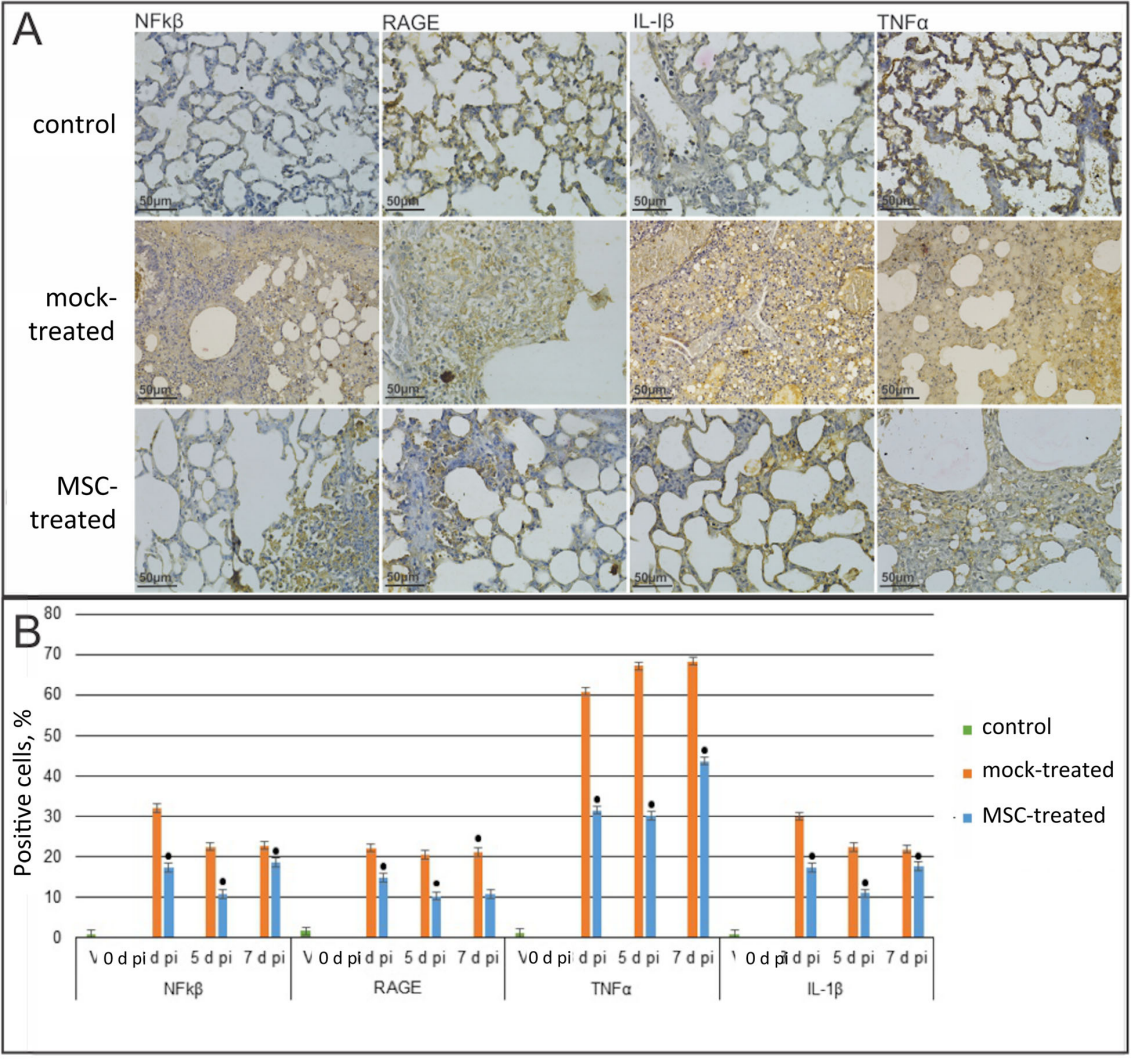
MSCs suppressed the expression of pro-inflammatory signaling proteins. **a** Immunohistochemical comparison of NF-kB, RAGE, TNFα, and IL-1β expressions in lung tissue between MSC- and mock-treated groups. Brownish color stains on the cell nucleus were observed under light microscopy with a ^X^400 magnification, indicating positive expressions of the proteins. **b** Quantitative comparison; the percentage of positive cells was determined by observation of 5 viewing fields for each tissue section. Data shown are means ± SD of 6 mice. ● indicates significant difference (P < 0.05) between two groups. d pi: days post-inoculation

We measured the protein levels of NF-kB, RAGE, TNFα, and IL-1β in lung tissue and we found that positive expression levels were induced by the A/H5N1 lethal infection (Fig. 6a, mock-treated groups). As shown in Fig. 6b, immunohistochemical analysis indicated that the expression levels of four proteins were attenuated significantly (*p* < 0.05) by the MSC treatment. The suppression rates were 50 to 55% on day 5.

### MSCs enhanced regeneration of alveolar epithelial cells in the lungs of mice with the a/H5N1 lethal infection

**Fig. 7:**
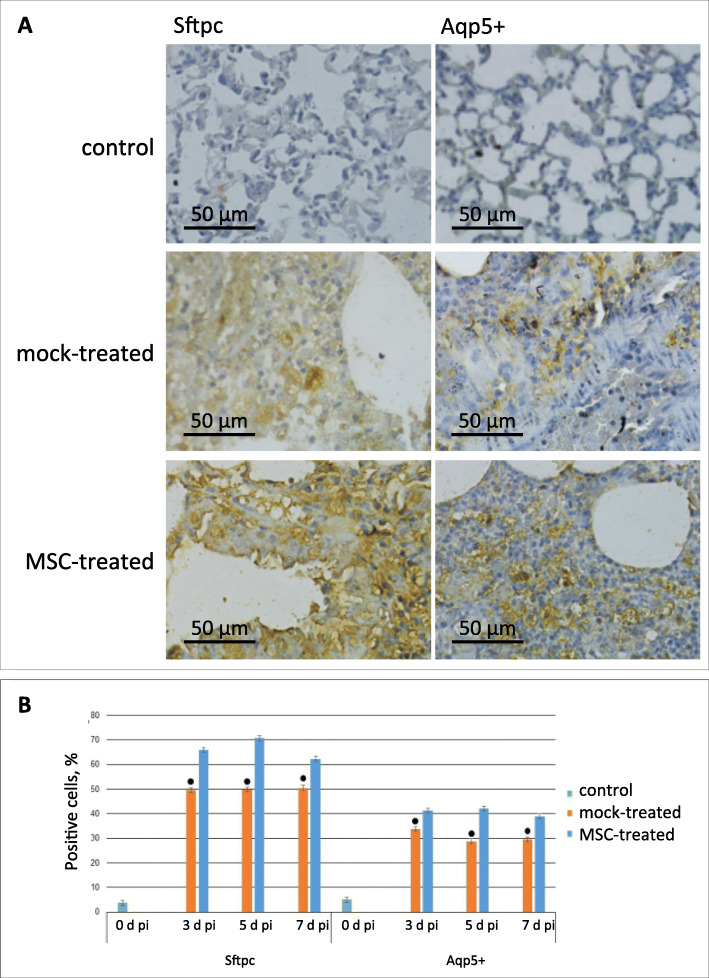
MSCs enhanced regeneration of alveolar epithelial cells. **a** Immunohistochemical comparison of the expression of Sftpc and Aqp5+ in lung tissue between MSC-treated and mock-treated groups on day 7. There were observed brownish color stains on the cell nucleus under a light microscopy with a ^X^400 magnification, indicating positive expressions of the proteins. **b** Quantitative comparison of the expressions; the percentage of positive cells was determined by observation of 5 viewing fields for each tissue section. Data shown are means ± SD of 6 mice. ● indicates significant difference (P < 0.05) between two groups. d pi: days post-inoculation

We measured for the expression of Sftpc as a marker of type II alveolar epithelial cell-progenitor, and Aqp5+ as a marker of type I alveolar epithelial cell-progenitor, by immunohistochemical examinations. There was obvious expression of both markers in the lung tissues of mice treated with the A/H5N1 virus and the expression levels increased significantly (p < 0.05) by MSC administration (Fig. 7a and b). The expression-enhanced rates on day 5 were 42 and 46% for Sftpc and Aqp5+, respectively.

### MSCs did not reduce viral proliferation in mice with the a/H5N1 lethal infection

**Fig. 8:**
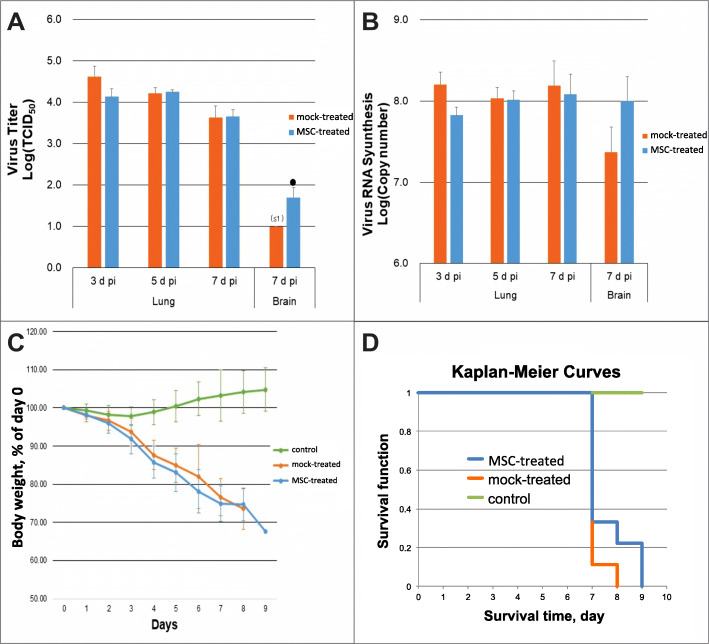
MSCs did not significantly affect viral proliferation, mouse morbidity, and mortality. **a** Infectious virus titers in lungs and brains of MSC- and mock-treated groups. Viral titers were expressed as the mean Log TCID_50_ per tissue ± standard error of 6 mice. **b** Comparison of viral RNA synthesis between MSC- and mock-treated groups by RT-PCR. The amounts of viral RNA synthesis were expressed as log (mean copy number per tissue) ± standard error. d pi: days post-inoculation. **c** The body weights of mice in MSC-treated D2–4-6 subgroup, mock-treated D2–4-6 subgroup, and control group: daily body weights (% of day 0) ± standard error. **d** The survival rates of mice in MSC-treated D2–4-6 subgroup, mock-treated D2–4-6 subgroup, and control group. The survival rates were analyzed by Kaplan-Meier curves. There was no significant difference between the two subgroups

To access the anti-viral effects of the MSC treatment, we examined for virus titers in the lung and brain of mice infected with the A/H5N1 virus. The means of virus titer and level of viral RNA synthesis in the MSC-treated group compared to the mock-treated group were not significantly different on days 3, 5, and 7 (Fig. 8, A and B). MSC treatment failed to inhibit virus proliferation. Furthermore, the virus proliferation in brain of the MSC-treated group was actually higher compared to that of the mock-treated group on day 7 (Fig. 8a and b). The infectious virus was detected only in brain of the MSC-treated group, and the means of viral RNA synthesis in brain of the MSC-treated group were 6-fold higher than those of the mock-treated group. The bodyweight of mice in MSC-treated D2–4-6 and mock-treated D2–4-6 subgroups began to decrease on day 2 compared to that of control group (Fig. 8c). The reduction was not significantly different between the two subgroups. The mortality rate was also not significantly different between them, although the survival rate of the MSC-treated subgroup was higher than that of the mock-treated subgroup on days 7 and 8; the number of survival mice was 3 on day 7 and 2 on day8, out of 10 for the MSC-treated D2–4-6 subgroup, while it was only 1 on day7 for the mock-treated D2–4-6 subgroup (Fig. 8d).

## Discussion

The beneficial effect of MSCs comes from their capacity to secrete paracrine factors that modulate immune response and change the response of epithelium, endothelium, and inflammatory cells to the injury. The administration of MSC allotransplantation begins with homing to the damaged alveolar epithelium of the lung. Homing of exogenous MSCs occurs due to signal interactions between exogenous MSCs and damaged lung tissue. In damaged lung tissue, phagocytic cells will produce the inflammatory mediators such as TNFα, IL-1β, free radicals, chemokines, and leukotrienes in excessive amount that will change the microenvironment. Then, mobilization and differentiation of MSCs occur, which in turn will replace cells from damaged tissue [46].

We demonstrated in this study that MSC homing occurred in lung tissue within 24 h after the administration of MSCs (Fig. 3). As a consequence, the progress of lung damage was prevented by day 1 day after MSC administration to the virus-infected mice; this was observed on days 3, 5, and 7, by macroscopic observation of the whole lung tissue (Fig. 4c), measuring lung alveolar protein (Fig. 4a), PaO_2_/FiO_2_ ratio (Fig. 4b), and histopathological score (Fig. 5b). A suppression of inflammation markers in line with the prevention of the lung injury was also observed (Fig. 6). MSC administration suppressed RAGE and NF-κB expressions and, as a consequence, caused decreased expression of inflammatory cytokines, TNFα and IL-1β. Our results were consistent with several previous reports. An in vivo study of mice infected with highly pathogenic avian influenza A/H5N1, conducted by Chan et al.*,* in 2016, proved that the administration of MSCs caused significantly lower levels of cytokines and chemokines [8]. Based on the research using mice with acute lung injury caused by avian influenza A/H9N2 virus, Li et al., and Yan et al.*,* concluded that MSC administration could reduce cytokine levels in the BAL specimens [11, 12]. However, these results were not consistent with the findings of Gotts et al., who reported that mice were unresponsive to MSC therapy; the administration of MSCs into mice infected with human influenza virus A/H1N1 Puerto Rico/8/34 (PR8) failed to reduce inflammation markers [13]. The conflicting results for the role of MSCs may be due to differences in lung injuries caused by the virus strains used.

In the present study, we demonstrated that infection with the highly pathogenic avian influenza A/H5N1 virus stimulated the expressions of Sftpc and Aqp5+, and that administration of MSCs enhanced both of these expressions (Fig. 7). Sftpc is a specific surface marker to type II alveolar epithelial cells and Aqp5+ to type I [47, 48]. In ARDS conditions, most layers of type I alveolar epithelial cells on the alveolar surface are damaged because of its extensive surface and complex branching architecture. Type I alveolar epithelial cells are unable to multiply through the mitosis process, either during lung growth or injury [48]. Stem cells or progenitor cells are located in the alveolar niche consisting of type I and II alveolar epithelial cells [29]. It is very likely that the homing MSCs can stimulate differentiation of progenitor cells into type II and I alveolar epithelia cells.

Body weight, virus growth, and survival period of mice with the A/H5N1 lethal infection were not changed significantly by MSCs administration in this study (Fig. 8). The virus presents multiple basic amino acid residues on HA cleavage sites (GISAID accession number: EPI1215564) causing HA to be broken down by many intracellular protease enzymes such as furin-like protease, so that it can cause systemic infections [49, 50]. The occurring death may be caused not only by lung injuries but also by the injuries of other organs. The A/H5N1 virus infection occurs in multi organs including the brain [51, 52] where it is difficult for MSCs to reach by intravenous administration [53, 54]. In accordance with their results, we also detected viral RNA synthesis in the brain for the mock-treated group with virus infection in this study (Fig. 8b). MSC-treatment increased viral RNA synthesis and the amount of the infectious virus in the brain (Fig. 8a, b), which explains why mice in the MSC-treated group died roughly at the same rate as that of the mock-treated group inspite of obvious suppression of lung injuries (Fig. 8d). A paper published 65 years ago showed influenza virus proliferation was inhibited in hypoxic mice and chicken embryos [55]. Our present study showed that the PaO_2_/FiO_2_ ratio lowered in mice with the A/H5N1 infection and that the MSCs treatment improved the ratio (Fig. 4b). This suggests that the virus infection causes hypoxia in the brain resulting from the lowered PaO_2_/FiO_2_ ratio in the blood and the hypoxia inhibits virus proliferation in the brain. The MSC treatment attenuates hypoxia as the ratio is improved. As a consequence, the inhibition is attenuated in the MSC-treated mice. Actually, we observed the attenuation of inhibition, i.e., increase of virus proliferation in brain in the MSC-treated mice (Fig. 8a, b). In the lung, the virus proliferates in alveolar and bronchiolar epithelial cells. Because of exposure to air, these cells do not become hypoxia with the virus infection even when hypoxia in blood. Therefore, there was no difference in lung for virus proliferation between MSC-treated and mock-treated mice (Fig. 8a, b).

Our primary interest in this study was the impact of MSC administration on the lung injuries caused by highly pathogenic avian influenza virus, which was most clearly shown by using the lethal infection dose. Future studies should investigate the potential therapeutic effects on mortality with different settings of virus doses, different schedules of MSC administration, combination use of anti-viral drugs or using non-brain tropic viruses. Our findings have important implications for clinical translational studies investigating MSC therapy for management of ALI and ARDS.

## Conclusions

The administration of MSCs prevented further lung injuries and inflammation, and enhanced alveolar cell type II and I regeneration, while it did not significantly affect viral proliferation and mouse morbidity and mortality. The results suggest that MSC administration is a promising strategy for treatment of acute lung injuries caused by the highly pathogenic avian influenza A/H5N1 virus, although further optimization and combination use of anti-viral drugs will be obviously required to achieve the goal of reducing mortality.

## Data Availability

The datasets used and/or analyzed during the current study available from the corresponding author on reasonable request.

## Abbreviations

ALI: Acute lung injury
ARDS: Acute respiratory distress syndrome
Aqp5+: Aquaporin 5
BALF: Bronchoalveolar lavage fluid
DAMPs: Danger associated molecular patterns
FITC: Fluorescein isothiocyanate
HE: Hematoxylin-eosin
HMGB1: High mobility group box 1
IL-Iβ: Interleukin─Iβ
MLD_50_: 50% mouse lethal dose
MSCs: Bone marrow-derived mesenchymal stem cells
NF-κB: Nuclear factor-κB
PAMPs: Pathogen-associated molecular patterns
PBS: Phosphate buffered saline
d pi: Days post inoculation
PRRs: Pattern recognition receptors
RAGE: Receptor for advanced glycation end products
Sftpc: Surfactant protein-C
TNF-α: Tumor necrosis factor-α
TGS: Tris buffered glucose-containing saline
